# Measurement of Crystalline Silica Aerosol Using Quantum Cascade Laser–Based Infrared Spectroscopy

**DOI:** 10.1038/s41598-017-14363-3

**Published:** 2017-10-24

**Authors:** Shijun Wei, Pramod Kulkarni, Kevin Ashley, Lina Zheng

**Affiliations:** 1 Centers for Disease Control and Prevention, National Institute for Occupational Safety and Health, Cincinnati, Ohio 45226 USA; 2 University of Cincinnati, Department of Mechanical and Materials Engineering, Cincinnati, Ohio 45221 USA

## Abstract

Inhalation exposure to airborne respirable crystalline silica (RCS) poses major health risks in many industrial environments. There is a need for new sensitive instruments and methods for in-field or near real-time measurement of crystalline silica aerosol. The objective of this study was to develop an approach, using quantum cascade laser (QCL)-based infrared spectroscopy (IR), to quantify airborne concentrations of RCS. Three sampling methods were investigated for their potential for effective coupling with QCL-based transmittance measurements: (i) conventional aerosol filter collection, (ii) focused spot sample collection directly from the aerosol phase, and (iii) dried spot obtained from deposition of liquid suspensions. Spectral analysis methods were developed to obtain IR spectra from the collected particulate samples in the range 750–1030 cm^−1^. The new instrument was calibrated and the results were compared with standardized methods based on Fourier transform infrared (FTIR) spectrometry. Results show that significantly lower detection limits for RCS (≈330 *ng*), compared to conventional infrared methods, could be achieved with effective microconcentration and careful coupling of the particulate sample with the QCL beam. These results offer promise for further development of sensitive filter-based laboratory methods and portable sensors for near real-time measurement of crystalline silica aerosol.

## Introduction

Inhalation exposures of workers to respirable crystalline silica (RCS) occur in a variety of occupations owing to its ubiquitous presence and the plethora of applications for silica-containing materials and minerals^1,2^. Inhalation of RCS can lead to silicosis and lung cancer, and has also been linked to chronic obstructive pulmonary disease, kidney disease, and autoimmune disorder^1,2^. High RCS exposures can be found in many industrial atmospheres and activities including cement, glass and ceramic production, semiconductor fabrication, foundries, sandblasting, construction activities, mining, and oil and gas drilling^1–4^. It has been estimated that more than 2 million workers are exposed to respirable crystalline silica across various industries in the United States^5^. In most workplaces RCS appears in the form of *α*-quartz or cristobalite, particularly as the former polymorph^1,2^. Several epidemiologic studies indicate that the current occupational standards for crystalline silica are not sufficiently protective to prevent chronic silicosis^1,2,5^.

Current standardized methods for measuring airborne RCS employ filter-based collection using a size-selective sampling device for collecting the respirable fraction^6^ of aerosol, followed by laboratory analysis using either X-ray diffraction (XRD)^7^ or infrared (IR) spectrometry^8^. Sample preparation procedures for RCS measurement differ depending on the analytical measurement technique used. Estimated method detection limits (MDLs) for RCS by XRD and IR range from 1 to >10 *μg* per sample^7,8^. Recent studies using new FTIR instrumentation^9^ and Raman microscopy^10^ have demonstrated LODs below 1 *μg*. In consideration of lower occupational exposure limits (OELs), better MDLs are desired. When sampling at airborne RCS concentrations below OELs such as the NIOSH Recommended Exposure Limit (REL)^11^, typical RCS sampler loadings are about 30 *μg* (and lower) and the measurement uncertainty of existing methods might not meet desired accuracy criteria^12^.

Currently used standardized methods for sampling and analysis of RCS by IR and XRD suffer from several drawbacks, including inconsistent analytical figures of merit at levels below OELs of interest and large sample collection times^13^. These filter-based methods were mainly developed for regulatory compliance measurement, rather than transient monitoring of worker exposures, and they may not be capable of capturing the short-term but high RCS exposures typical in many construction, mining, and other industrial activities^14^. Many non-routine investigational or surveillance applications require measurements below existing occupational exposure limits. There remains a demand for short measurement times (on the order of minutes) to capture acute, high-intensity exposures to promote quick, on-site decision making for effective hazard identification and mitigation.

In this study, we present a new technique, using quantum cascade laser (QCL)^15^ -based infrared spectroscopy, with applications to in-field or near real-time measurement of airborne RCS. QCLs are solid-state semiconductor lasers offering tunable coverage across the mid-IR range and are seeing myriad applications for sensing trace chemical agents^16^. QCL source intensities are much greater than those attainable using conventional blackbody radiative (globar) IR sources, thereby offering significant advantages for improving analytical sensitivity^17^. In addition, QCLs offer analytical dynamic ranges over several orders of magnitude. Moreover, their miniature size and robust performance makes them suitable for field portable instrumentation. Their applications in atmospheric chemistry have been exploited^18–22^. In this work we demonstrate, for the first time, the use of this microspectroscopy technique for measurement of trace levels of *α*-quartz aerosol. Quantitative QCL measurement of RCS is demonstrated for masses exceeding 350 *μg* per sample, and MDLs <1 *μg* per sample have been attained.

## Methods

### Experimental Setup

**Figure 1:**
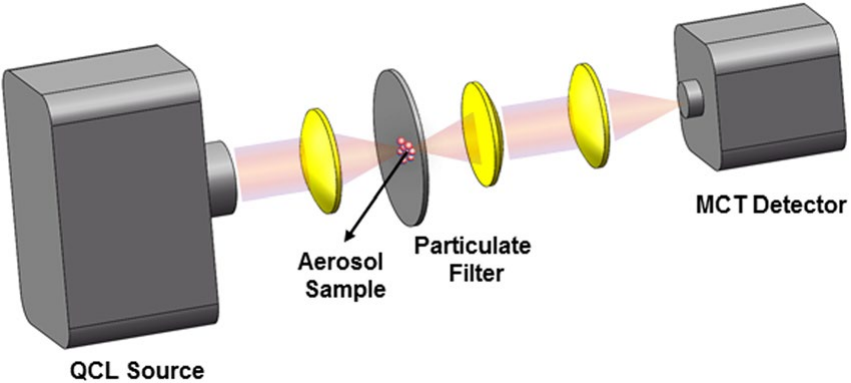
Instrument setup used for filter transmittance measurement using QCL source and MCT detector (not to scale).

The measurement system used in this work consisted of a QCL source (LaserTune, Block Engineering, Marlborough, MA, USA), beam-shaping optics, filter sample holder, signal collection optics, and a mercury-cadmium-telluride (MCT) IR Detector Module (Block Engineering). The QCL was configured to operate over the wavenumber range of 750–1030 cm^−1^ (using an external cavity). It would be desirable to extend the wavenumber range down to 650 cm^−1^ (to account for spectral interferences from certain constituents such as kaolinite); however, such QCL sources are not yet commercially available. IR-transparent convex lenses (ZnSe; Thorlabs, Newton, NJ, USA) were used to shape the IR beam to a circular cross sectional profile and achieve focus at the surface of the membrane filter containing collected particulate matter. Two lenses were positioned behind the membrane filter to collect the transmitted infrared radiation and focus it directly on the IR-sensitive surface of the MCT detector. The assembly was mounted on a vertical track rail, allowing for fine adjustment of the distance between the optical components and optimization of the alignment to maximize the measured transmittance signal. Figure 1 is schematic diagram showing the arrangement of the QCL source, membrane filter sample and the MCT detector.

The QCL was operated in scanning mode to allow rapid variation of source frequency in the spectral range of interest. Signal acquisition of the MCT detector was synchronized with the QCL scanning to allow reconstruction of IR spectra in the range of 750–1030 cm^−1^. The respirable quartz peak was measured at 798 cm^−1^ in accordance with established IR spectrometric practice^8^.

### Sample Preparation

**Figure 2:**
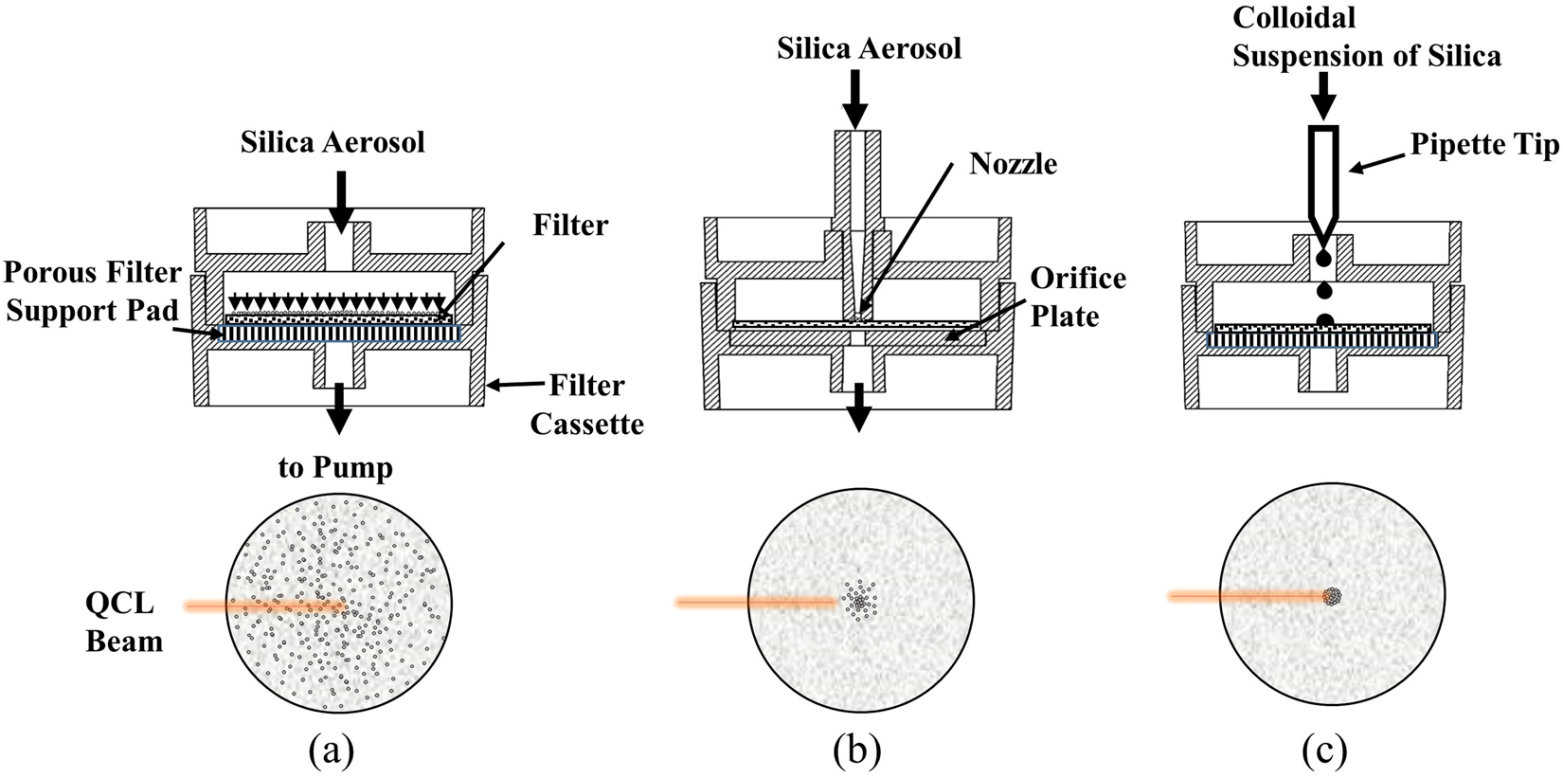
Three different particle collection methods used in this study: (**a**) method A: conventional filter collection; (**b**) method B: direct aerosol microconcentration using a nozzle, and (**c**) method C: dried spot obtained using micro-aliquots of colloidal supsension. The filter schematics illustrate the spatial extent of the collected particulate deposit with respect to the size of the filter and QCL beam.

Particulate filter samples used for QCL-IR analysis were prepared using three separate methods shown in Fig. 2. These methods include: (i) conventional filtration involving particle collection over the entire filter surface (Fig. 2a, method A), (ii) focused collection over a very small spot (<1 mm diameter) using a converging nozzle (Fig. 2b; method B); and dried spot obtained using micro-aliquots of colloidal silica suspension (Fig. 2c; method C). For method A and method C, a 25-mm diameter filter was housed in a 25-mm filter cassette (SKC Inc., Eighty-Four, PA, USA) as shown in Fig. 2. For method B, a 37-mm dia. filter was housed in a 37-mm filter cassette (SKC Inc.). Three types of filters were investigated to ensure that there was no spectral interference in the wavenumber range of interest: Polyvinyl chloride (PVC), polycarbonate (PC) and polypropylene (PP) filters, 37- and 25-mm in diameter (SKC Inc.). Certified standard reference filters with known RCS loadings, developed by the Workplace Analysis Scheme for Proficiency program (WASP; Health and Safety Laboratory, Buxton, UK) for quality control and proficiency testing, were also used for calibration and testing purposes. These certified filter samples are prepared in accordance with the ISO Guide 33^23^.

For methods A and B, test aerosol was generated by aerosolizing aqueous suspensions of Standard Reference Material respirable quartz (SRM 1878a; NIST, Gaithersburg, MD, USA) using a Collison nebulizer (BGI, Butler, NJ, USA), and subsequently drying the aerosol with a diffusion dryer (TSI Inc., Shoreview, MN, USA). In method A (Fig. 2a), the particulate filter was supported by a porous cellulosic support pad (as is conventional), while in method B it was supported using a nonporous disc with a center orifice (0.8 mm diameter). The center orifice helped reduce expansion of the aerosol flow jet exiting the nozzle and achieved a smaller spot size of the particulate deposit. The total particulate mass loadings in methods A and B were obtained using the measured aerosol mass concentration at the inlet of the cassette and the total collection time. The mass concentration of the resulting dried aerosol was measured using a DustTrak instrument (Model 8533, TSI Inc.). The DustTrak was calibrated with respect to gravimetric measurements (to +/− 0.001 mg) using the same test aerosol to allow accurate estimation of filter mass loadings.

Method C involves depositing an aliquot of known volume (few microliter) from a liquid suspension onto a filter substrate and subsequently allowing the liquid to dry into a concentrated particulate residue—the ‘dried spot’. The resulting dried spot is then analyzed using QCL-IR. Similar dried spot methods have been successfully used before for other microspectroscopies^24^. To generate a calibration curve for method C, suspensions of quartz SRM were first prepared in ultrafiltered deionized water (CAS 7732-18-5, ThermoFisher Scientific, Rochester, NY, USA) with various predetermined aqueous concentrations ranging from 0.2 to 6 mg/ml. A 1.5 *μl* aliquot of this suspension was then deposited at the center of the 37 mm PVC filter, which resulted in a spot size <2 mm diameter. The filters were subsequently air dried to obtain the dried spot sample.

The QCL was operated in scanning mode to allow rapid variation of source frequency in the spectral range of interest. Signal acquisition of the MCT detector was synchronized with the QCL scanning to allow reconstruction of IR spectra in the range of 750–1030 cm^−1^. The respirable quartz peak was measured at 798 cm^−1^ in accordance with established IR spectrometric practice^8^. For purposes of comparison with a standardized IR method, WASP samples were measured by both the QCL-IR approach and a NIOSH IR procedure, method 7603^25^ by means of FTIR spectrometry (Model Spectrum 1000; Perkin Elmer, Waltham, MA, USA). NIOSH method 7603 is harmonized with the redeposition sample preparation followed by IR analysis methodology of ASTM D7948^8^. FTIR measurements involved placing the as-received WASP filter sample directly in the path of the beam, followed by transmittance measurements as described in NIOSH method 7603. A blank WASP filter sample (with no silica mass loading) was used to obtain a reference spectra. The QCL-IR measurements were obtained similarly (on the same WASP filter sets) by placing the filter sample in the path of the QCL beam. Since the diameter of the FTIR and QCL beams were different, the actual mass of analyte irradiated by the beam in each method was different (the mass sampled by FTIR was more by approximately two orders of magnitude, assuming uniform analyte density on the WASP filter).

### Spectral Analysis

The measured QCL-IR spectra were analyzed using two methods to quantify the content of crystalline silica in the particulate sample. The first spectral analysis method was a univariate approach involving measurement of peak height at 798 cm^−1^. A straight line connecting the transmittance values at 766 cm^−1^ and 842 cm^−1^ was used as a baseline. The baseline transmittance at 798 cm^−1^ was then subtracted from the peak transmittance at 798 cm^−1^ to obtain the corrected signal. The measured transmittance signal was proportional to the crystalline silica content.

The second spectral analysis method involves a multivariate technique using partial least square (PLS) regression of the entire spectrum^26^. The PLS model involves linear regression between observable variables **X** and predictor variables **Y** in a new space, such that:

1 $${\bf{X}}={\bf{T}}{{\bf{P}}}^{T}+{\bf{E}}$$

2 $${\bf{Y}}={\bf{U}}{{\bf{Q}}}^{T}+{\bf{F}}$$

where **T** and **U** matrices are the projections of **X** and **Y**, **P** and **Q** matrices are loadings of the original variables, and **E** and **F** are the residual matrices which are related to the noise. The transmittance data from QCL measurements in the wavenumber range 766–812 cm^−1^, containing 24 pixels, were used as **X** variables, while 14 filter samples with mass loadings ranging from 5.4–48.9 *μg* were used as **Y** variables. The spectral data were pre-processed using an auto-scaling and mean-centering method. Two latent variables were sufficient to build the model and provided the smallest root mean square error. The ‘leave-one-out’ cross validation method^27^ was used to test the reliability of the PLS model when used to predict the unknown samples. The method detection limit, MDL (*m*_*mdl*_), of RCS using the PLS model was estimated as^28,29^:

3 $${m}_{mdl}=3.3{\delta }_{\chi }||{\bf{b}}||$$

where *δ*_*χ*_ is an estimate of the noise level in the data (obtained by measuring the variation of the noise in the selected wavenumber region) and ||**b**|| represents the Euclidian norm of the vector of regression coefficients.

## Results and Discussion

**Figure 3:**
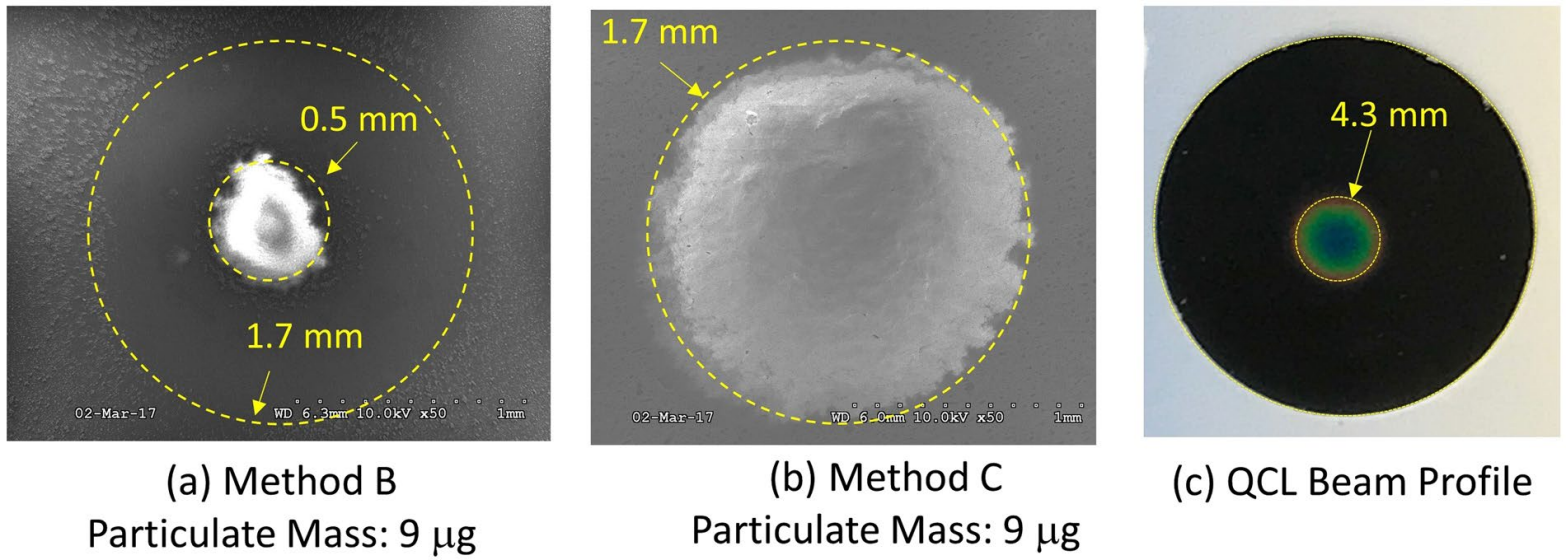
Spatial extent of the particulate deposits on filters using (**a**) method B, (**b**) method C, and (**c**) the QCL beam spatial profile seen on an IR-sensitive card. The diameter of the IR-illuminated area on the sensor card was ≈4.3 mm; however, the QCL beam diameter (relevant for transmittance measurement) was estimated to be ≈1.5 mm.

Figure 3a-b shows images obtained from scanning electron microscopy of particulate deposits using methods B and C. Method B, involving direct aerosol collection via a nozzle, leads to a nearly circular deposit in the filter center containing most of the RCS sample mass, with an approximate diameter of 0.5 mm. A closer look at the micrograph revealed that the particles were also collected beyond an outer diameter of 1.7 mm (Fig. 3a). Very few particles deposit between 0.5 mm and 1.7 mm. This region was masked by the nozzle walls, whose thickness was approximately 1 mm. This smeared collection of particles is likely attributable to expansion of the flow at the nozzle exit, resulting in collection over a wider area. On the other hand, the dried spot using method C has a diameter of 1.7 mm; it is well defined and does not show any smear. Figure 3c shows the spatial extent of the QCL beam obtained using a mid-IR sensor card (CAS#: 7758-02-3, International Crystal Labs, Garfield, NJ). The beam diameter was large enough to illuminate most of the collected particles in methods B and C.

**Figure 4:**
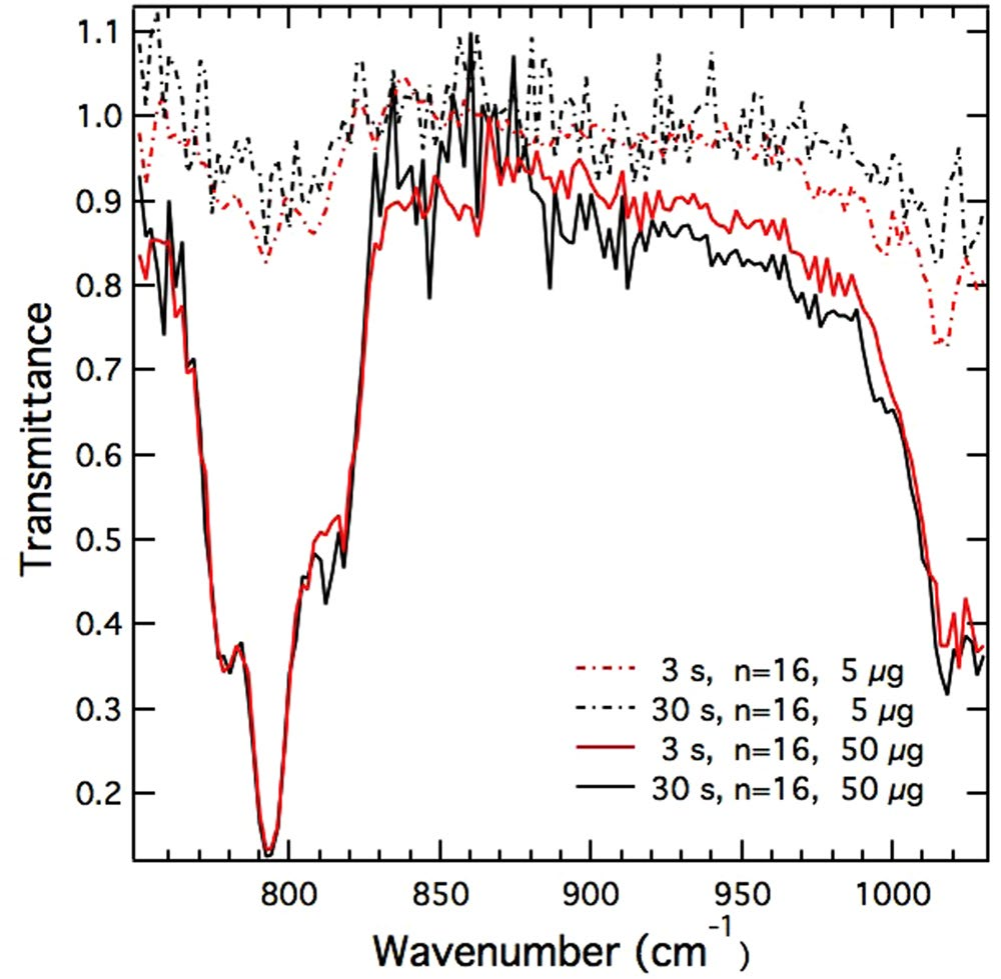
QCL-IR spectra of NIST SRM 1878a quartz on polycarbonate (PC) filters at mass loadings of ≈5 and ≈50 *μ*g per filter.

To investigate the effect of filter media on the analyte signal, QCL-IR spectra were measured using three types of filters for given particulate mass loadings: PVC, PC, and PP. PVC and PC filters were found to provide better signal-to-noise ratio compared to PP filters and were subsequently used in all ensuing experiments. Detector integration time and spectral averaging were optimized to obtain maximum signal-to-noise ratio. Figure 4 shows QCL-IR spectra of quartz SRM deposited on PC filters at two mass loadings (5 *μg* and 50 *μg*) at different integration times. The peaks at 780 cm^−1^ and 798 cm^−1^ are attributed to the doublet (ascribed to *α*-quartz Si stretching modes: ←*Si*–*O*–*Si*→) that has been widely used for analytical detection and quantification of RCS from workplace samples using conventional IR spectrometry^8,25^. The spectra show increased noise below 768 cm^−1^, attributed to reduced QCL source irradiance and detector responsivity in this spectral range. The relative standard deviation of repeat scans at a given wavenumber below 768 cm^−1^ was in the range 1.8–8.5%, while it was 0.3–1.3% in the wavenumber range 768–826 cm^−1^. Integration times greater than 30 s did not show improvement in signal-to-noise ratio. Most spectra in this study were obtained with 3 s scan time and 16 co-adds.

**Figure 5:**
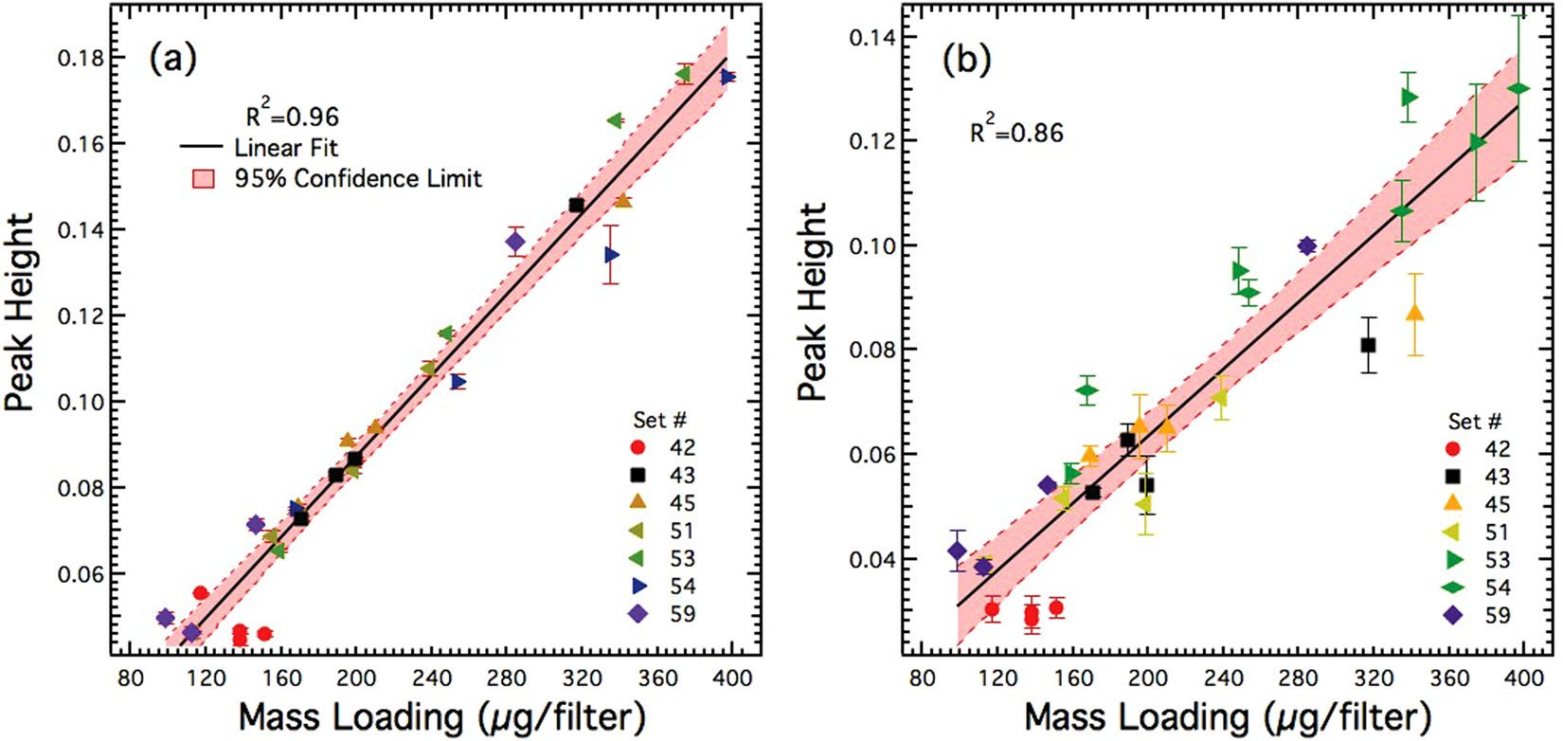
Measured peak transmittance of WASP certified filter samples using (**a**) QCL-IR and (**b**) FTIR at ≈800 cm^−1^.

Measurements were carried out on certified WASP filter samples to obtain calibration curves using the FTIR method as well as the QCL-IR technique. Figure 5a shows peak signal (proportional to transmittance) vs. analyte mass per filter from the QCL measurements of WASP filter samples; Fig. 5b shows similar measurements from the FTIR method on the same filter samples. The peak signal was calculated using the univariate approach described earlier. Each data point in Fig. 5 represents a mean of at least 3 repeat measurements of the same filter, and the error bar represents the standard deviation around the mean. Each FTIR measurement represented the average of four scans, with each scan obtained by successively rotating the filter by 45^o^ (as described in NIOSH method 7602^30^). Each data point for QCL-IR measurement represents an average over three measurements, with each measurement obtained at three random locations within a radius of 5 mm of the filter center. Total signal from QCL measurements show a strong correlation with the corresponding nominal/certified mass loadings of WASP filter samples.The range of relative standard deviation (RSD; including the minimum and maximum) in Fig. 5a and b was 0.2–5% (0.2–3% excluding one outlier) and 1–11.8%, respectively. The *R* ^2^ for linear regression in Fig. 5a was 0.96, while that in Fig. 5b was 0.86. QCL measurements clearly show better precision compared to that of the FTIR.

**Figure 6:**
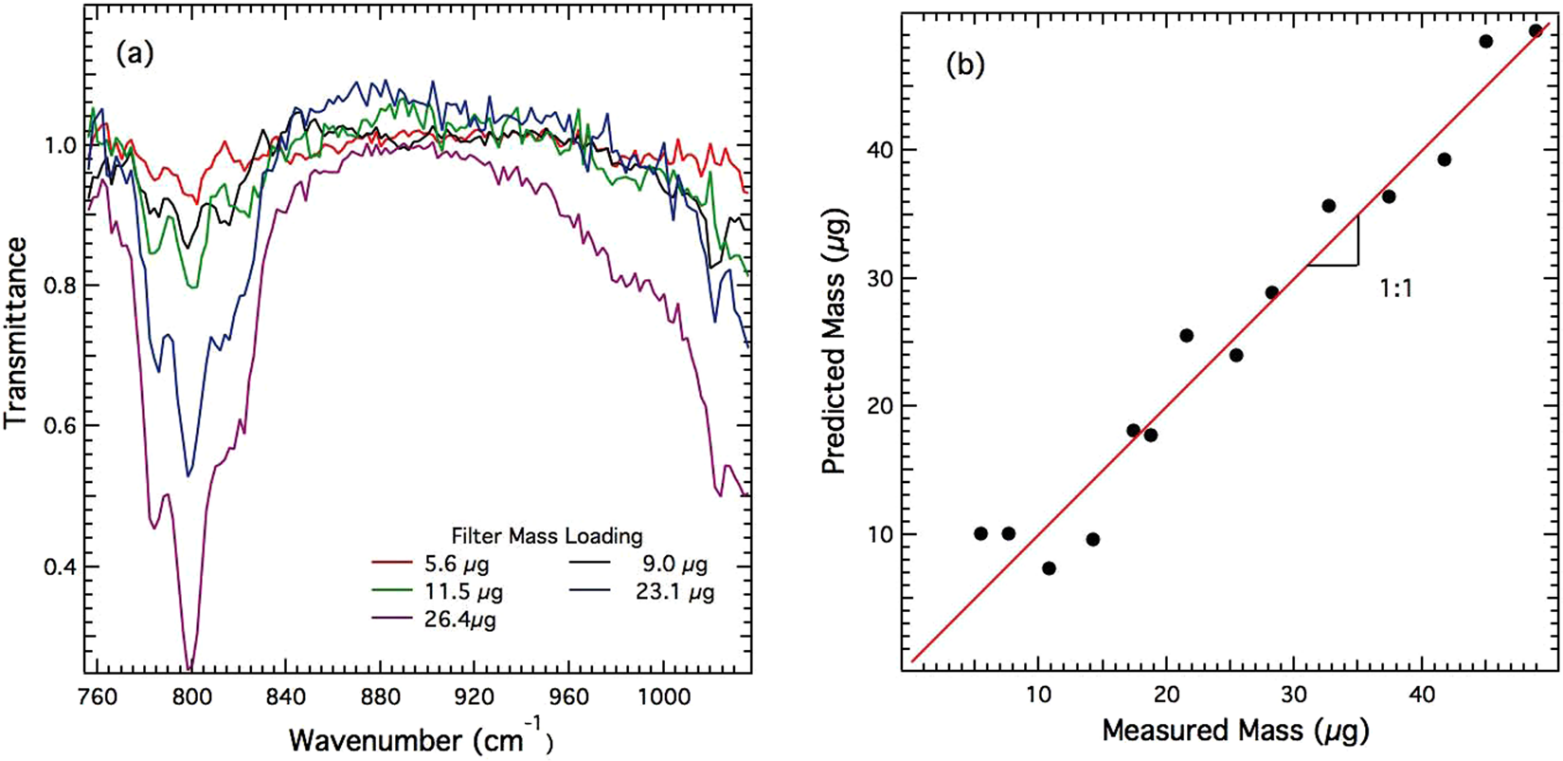
(**a**) QCL-IR spectra of samples prepared using method B for samples with RCS mass loadings ranging from 5.6 to 26.4 *μ*g; (**b**) corresponding plot showing cross validation performance of the PLS regression model.

Figure 6a shows IR spectra obtained from filter samples prepared using sample preparation method B for mass loadings ranging from 5.6 to 26.4 *μg*. These spectral data were then used to construct a PLS calibration model. Figure 6b shows predicted mass using this PLS calibration. Using Equation (3), the calculated MDL of method B was 3.3 *μg*. These measurements demonstrate the utility of QCL method for measurement of RCS at levels of interest for occupational exposure monitoring.

**Figure 7:**
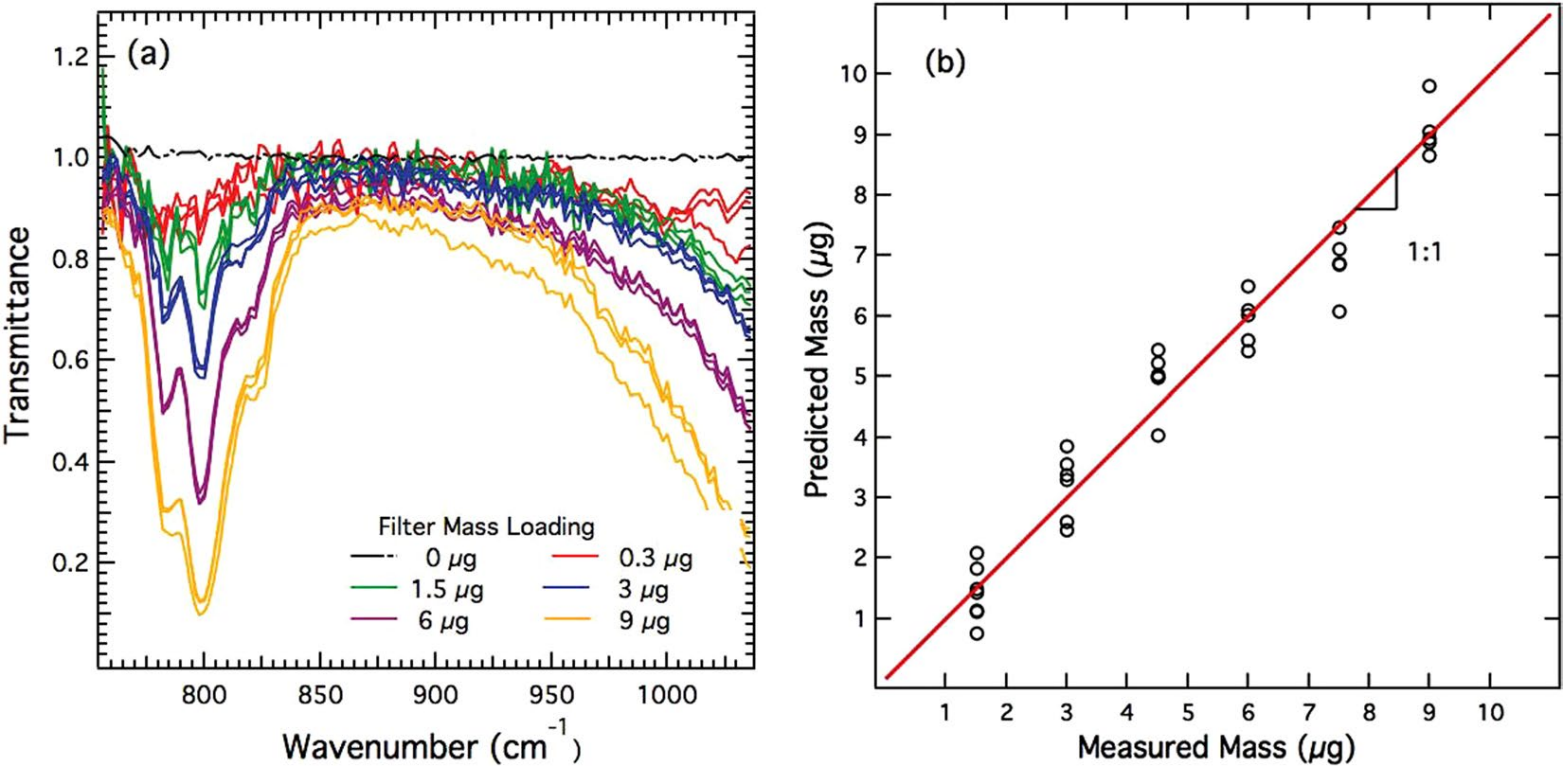
(**a**) QCL-IR spectra of samples prepared using method C for samples with RCS mass loadings <10 *μ*g; (**b**) corresponding plot showing cross validation of the PLS regression model.

To attain yet lower quartz loadings on filters, samples were prepared using method C, by pipetting aliquots from a suspension of quartz SRM suspended in water. The area of the circular quartz deposit (after allowing for evaporation of water) on the PVC filter was approximately 1.7 mm, as shown in Fig. 3b, and roughly matched the spatial extent of the QCL beam (Fig. 3b). PVC filters were used as substrate. These hydrophobic filters allowed drying of the aliquot droplet without spreading, preventing its distribution over wider filter area. QCL spectra obtained from PVC filters with quartz loading below 10 *μg* per filter are shown in Fig. 7a, with separate filters at each mass loading prepared in triplicate. Compared to method B, method C provides much lower detection limits, allowing quantitative RCS measurement of 1.5 *μg*. The repeatability of measurements is also superior when using the latter method. The measured spectra were used to construct a PLS calibration model, whose prediction performance is shown in Fig. 7b. The best fit to the predicted and measured mass in Fig. 6b is shown, with *R* ^2^ = 0.93. The calculated MDL obtained using Equation (3) was 0.3 *μg*.

The results of this work demonstrate that QCL-IR spectroscopy can be used to accurately measure trace RCS, and offer promise for short-term exposure monitoring applications. Each of the three aerosol collection methods studied offer unique advantages and disadvantages. Method A is the most widely used technique for workplace monitoring and offers the easiest access to QCL measurements, and is analogous to the current on-filter FTIR methods^31^. Although the MDL of this method for QCL measurement was not established in this study, we expect the measurement precision to be better than that of the FTIR measurement (Fig. 5). Method B requires a special nozzle to allow for concentrated collection of aerosol, and should provide improved detection limits compared to conventional filter collection in method A. However, we anticipate that the collection method used in this study can be further improved to reduce smearing of particle deposits seen in Fig. 3a. Improved microconcentration or focussing should further help reduce the MDL of this technique below 3.3 *μg*, perhaps by a factor of up to 10. Since method B involves direct aerosol collection followed by immediate analysis, it can be readily used in a real-time or direct-reading instrument. Method C offers the lowest MDL and demonstrates the superior measurement capability of QCL-based approach and can be coupled with different sample preparation methods. The low detection limits obtained using method C are also consistent with similar results obtained for other microspectroscopy techniques using the dried spot method^24^.

With respect to aerosol measurement in the infrared range, QCL-IR approach offers two unique advantages over traditional FTIR instrumentation: (i) miniature or compact size of the QCL source; and (ii) high source irradiance of QCLs. The miniature size allows effective or direct integration of QCLs with aerosol sampling devices or methods to conduct either substrate-based or *in-situ* aerosol measurements. The high source irradiance, on the other hand, allows using high path length configurations as, for example, in optical waveguides, multi-pass IR cells, or ring-down cavities to substantially improve detection limits or time resolution of measurements. As another example, high source intensity of the QCL can allow measurement using sequential stacking of multiple filters to enhance absorption and further improve detection limits. Our preliminary results suggest that the signal can be enhanced by a factor of approximately 1.6 when stacking two filters and by 2.6 with 3 stacked filters, compared to that from a single filter with identical analyte mass irradiated by the IR beam.

## Conclusions

QCL-IR measurements of certified RCS samples in the mass loading range of 100–350 *μg* per filter compared favorably to the FTIR measurements and showed superior precision in this study. QCL-IR measurements using microconcentration directly from the aerosol phase (method B) offer promise for further development of a near-real-time monitor for crystalline silica measurement. The estimated MDL of 3.3 *μg* of this method could be further reduced using more efficient microconcentration or aerosol focusing techniques. Finally, the manual method involving dried spot sample obtained from aliquots (method C) provides the best spectroscopic detection limits (≈0.3 *μg*) when the size of the dried spot is equal to or smaller than that of the QCL beam. This method also offers the ability to pretreat filter samples to minimize matrix interferences (for example, following protocols outlined in ASTM D7948); however such pretratment may increase detection limits beyond 0.3 *μg*. While recent studies^9,10^ have shown that new FTIR and Raman instrumentation can provide similar LODs reported here, QCL-based spectrometers offer unique advantages with respect to development of compact, hand-portable, near real-time aerosol sensors.

### Disclaimers

Mention of company names and products does not constitute endorsement by NIOSH. The findings and conclusions in this report are those of the authors and do not necessarily represent the views of NIOSH.

### Data Availability Statement

All key data generated or analyzed during this study are included in this published article.
